# Human Health Effects of Tetrachloroethylene: Key Findings and Scientific Issues

**DOI:** 10.1289/ehp.1307359

**Published:** 2014-02-14

**Authors:** Kathryn Z. Guyton, Karen A. Hogan, Cheryl Siegel Scott, Glinda S. Cooper, Ambuja S. Bale, Leonid Kopylev, Stanley Barone, Susan L. Makris, Barbara Glenn, Ravi P. Subramaniam, Maureen R. Gwinn, Rebecca C. Dzubow, Weihsueh A. Chiu

**Affiliations:** 1 National Center for Environmental Assessment,; 2 Office of Pollution Prevention and Toxics, and; 3 Office of Children’s Health Protection, U.S. Environmental Protection Agency, Washington, DC, USA

## Abstract

Background: The U.S. Environmental Protection Agency (EPA) completed a toxicological review of tetrachloroethylene (perchloroethylene, PCE) in February 2012 in support of the Integrated Risk Information System (IRIS).

Objectives: We reviewed key findings and scientific issues regarding the human health effects of PCE described in the U.S. EPA’s *Toxicological Review of Tetrachloroethylene (Perchloroethylene)*.

Methods: The updated assessment of PCE synthesized and characterized a substantial database of epidemiological, experimental animal, and mechanistic studies. Key scientific issues were addressed through modeling of PCE toxicokinetics, synthesis of evidence from neurological studies, and analyses of toxicokinetic, mechanistic, and other factors (tumor latency, severity, and background rate) in interpreting experimental animal cancer findings. Considerations in evaluating epidemiological studies included the quality (e.g., specificity) of the exposure assessment methods and other essential design features, and the potential for alternative explanations for observed associations (e.g., bias or confounding).

Discussion: Toxicokinetic modeling aided in characterizing the complex metabolism and multiple metabolites that contribute to PCE toxicity. The exposure assessment approach—a key evaluation factor for epidemiological studies of bladder cancer, non-Hodgkin lymphoma, and multiple myeloma—provided suggestive evidence of carcinogenicity. Bioassay data provided conclusive evidence of carcinogenicity in experimental animals. Neurotoxicity was identified as a sensitive noncancer health effect, occurring at low exposures: a conclusion supported by multiple studies. Evidence was integrated from human, experimental animal, and mechanistic data sets in assessing adverse health effects of PCE.

Conclusions: PCE is likely to be carcinogenic to humans. Neurotoxicity is a sensitive adverse health effect of PCE.

Citation: Guyton KZ, Hogan KA, Scott CS, Cooper GS, Bale AS, Kopylev L, Barone S Jr, Makris SL, Glenn B, Subramaniam RP, Gwinn MR, Dzubow RC, Chiu WA. 2014. Human health effects of tetrachloroethylene: key findings and scientific issues. Environ Health Perspect 122:325–334; http://dx.doi.org/10.1289/ehp.1307359

## Introduction

Tetrachloroethylene (perchloroethylene, or PCE) is a widely used chlorinated solvent that is produced commercially for use in dry cleaning, textile processing, and metal-cleaning operations. PCE has been detected in drinking water, indoor environments, ambient air, groundwater, and soil. Many point sources of contamination exist in the United States [U.S. Environmental Protection Agency (EPA) 2013c], and PCE is also commonly found at Superfund hazardous waste sites (U.S. EPA 2013b). Regarding exposure to the general population, the Centers for Disease Control and Prevention (2013) reported that PCE levels assessed in the most recent biomonitoring survey [the 2003–2004 subsample of the National Health and Nutrition Examination Survey (NHANES)] appeared to be similar or slightly lower than levels reported in earlier NHANES surveys. The primary exposure routes are via inhalation, including as a result of vapor intrusion from contaminated soil and water (U.S. EPA 2013d), and ingestion of contaminated water.

The U.S. EPA identified PCE as a priority existing chemical for regulatory action review under the Toxic Substances Control Act (U.S. EPA 2012c) and as one of several volatile organic compounds to be regulated as a group in drinking water (U.S. EPA 2010). Supporting these and other agency actions, the U.S. EPA Integrated Risk Information System (IRIS) program released an updated human health assessment of PCE in February 2012 that included an extensive toxicological review (U.S. EPA 2012b), hereafter referred to as the Toxicological Review. The Toxicological Review was developed according to the general guidelines for risk assessment set forth by the National Research Council (NRC 1983, 1994) as well as relevant U.S. EPA Guidelines and Risk Assessment Forum technical panel reports (U.S. EPA 2013a). The literature search strategy was based on the Chemical Abstracts Service Registry Number (CASRN) and at least one common name. Primary peer-reviewed literature published during or before August 2011 was included. Public submissions to the U.S. EPA and peer-reviewed information (including health assessments developed by other organizations, review articles, and independent analyses of the health effects data) were also considered for inclusion. Toxicokinetic, mechanistic, and other data (e.g., tumor latency, severity, background rate) were considered in interpreting experimental animal cancer findings. Individual study evaluation considered essential design features (particularly, the study species and its population and the relevance of the exposure paradigm); other considerations are discussed in the following sections. The Toxicological Review (U.S. EPA 2012b) provides additional detail regarding the source literature databases, the relevant U.S. EPA guidance, and the study evaluation criteria.

The assessment development involved multiple internal and external peer review stages. Critical input was provided by a 2004 peer consultation workshop on PCE neurotoxicity (U.S. EPA 2004), a 2010 NRC panel report (NRC 2010), a 2011 peer review of the physiologically based pharmacokinetic (PBPK) model applications (U.S. EPA 2012b), and written and oral comments from scientists within the U.S. EPA, other federal agencies, and the Office of Management and Budget (U.S. EPA 2012a) as well as the public (Regulations.gov 2008). Herein we describe key findings and scientific issues addressed in the U.S. EPA’s 2012 Toxicological Review of PCE, covering the following topics:

The role of metabolism in toxicity, informed by the development and application of an updated PBPK model
The carcinogenicity of PCE, based on analyses of epidemiological studies, multiple laboratory animal bioassays, and mechanistic data
Noncancer toxicity, focusing on neurotoxicity as a sensitive outcome.

## Role of Metabolism in PCE Toxicity

PBPK models can aid in integrating complex toxicokinetic information on the absorption, distribution, metabolism, and excretion of environmental chemicals and their metabolites. These models are constructed from physiologic information in addition to chemical- and metabolite-specific toxicokinetic data. Some models separate data sets for model calibration and evaluation, utilizing Bayesian methods to strengthen model predictions. PBPK models are used in human health assessments to predict the extent and nature of metabolism across species or exposure routes.

**Figure 1:**
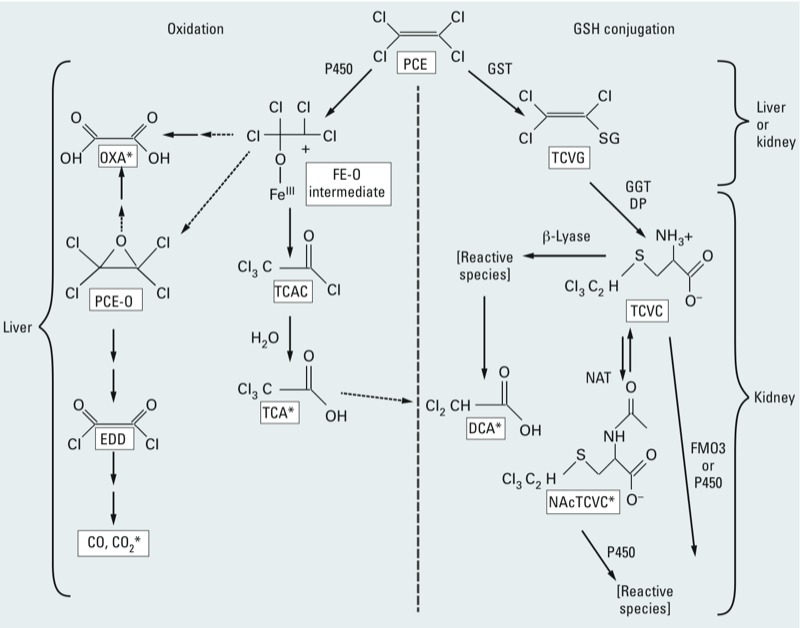
Simplified PCE metabolism scheme. PCE is metabolized in humans and experimental animal species by both oxidation (left) and GSH conjugation (right) metabolic pathways, yielding numerous toxicologically active compounds (Lash and Parker 2001). Tetrachlorethylene metabolism yields the oxidative metabolites TCAC, which hydrolyses to yield TCA, and the epoxide PCE-O, which decomposes in turn to EDD, CO, and CO_2_. OXA is also a product of PCE oxidation. GSH conjugation products include TCVG, the cysteine conjugate TCVC, and the mercapturate NAcTCVC and its sulfoxidation products. DCA is likely produced via β-lyase–mediated bioactivation, although TCA dechlorination may be an additional minor source. *Metabolites identified in blood, urine, or breath after *in vivo* PCE exposure (rodent or human).

The metabolism of PCE yields multiple metabolites through two main irreversible pathways: *a*) oxidation via the microsomal mixed-function oxidase system [i.e., cytochrome P450s (CYPs)], and *b*) conjugation with glutathione (GSH) by glutathione *S*-transferases (GSTs) (Lash and Parker 2001). Oxidation (Figure 1, left) occurs predominantly in the liver to a ferric-oxide (Fe-O) intermediate, the primary fate of which is thought to be trichloroacetyl chloride (TCAC), which then hydrolyses to yield trichloroacetic acid (TCA). A secondary fate of oxidation is the epoxide (PCE-O), which decomposes to ethandioyl dichloride (EDD) and then to carbon monoxide (CO) and carbon dioxide (CO_2_) (Yoshioka et al. 2002). Oxalic acid (OXA) has been reported as both an *in vivo* and *in vitro* product of PCE oxidation (Pegg et al. 1979; Yoshioka et al. 2002) and may be derived either from the epoxide or directly from the Fe-O intermediate. PCE conjugation with GSH (Figure 1, right) in the liver or kidney forms trichlorovinyl glutathione (TCVG), which is further processed by γ-glutamyl transpeptidase (GGT) and cysteinylglycine dipeptidase (DP) in the kidney, forming the cysteine conjugate *S*-trichlorovinyl-l-cysteine (TCVC). TCVC may be bioactivated by β-lyase or by flavin-containing monooxygenases (FMO3s) or CYP3A to reactive species (Anders et al. 1988; Krause et al. 2003), or (reversibly) may undergo *N*-acetylation [by *N*-acetyltransferase (NAT)] to the mercapturate *N*-acetyl trichlorovinyl cysteine (NAcTCVC). NAcTCVC is then excreted in urine or sulfoxidated by CYP3A to reactive species (Werner et al. 1996). Dichloroacetic acid (DCA), excreted in urine, is thought to be an end product of β-lyase–mediated bioactivation (Lash and Parker 2001), although a small contribution from TCA dechlorination cannot be ruled out.

PCE liver effects are thought to result from oxidative metabolites (Buben and O’Flaherty 1985), whereas metabolites resulting from GSH conjugation are hypothesized to cause kidney effects (Lash and Parker 2001). The identity of PCE metabolites involved in the induction of other PCE health effects is less clear, although PCE itself has been presumed to cause neurological effects (e.g., Boyes et al. 2009).

Many PBPK models for PCE have been developed to predict the relationship between external measures of exposure and internal dose [Bois et al. 1996 (updated by Chiu and Bois 2006); Chen and Blancato 1987; Chien 1997; Clewell et al. 2005; Covington et al. 2007 (updated by Qiu et al. 2010); Gearhart et al. 1993; Loizou 2001; Rao and Brown 1993; Reitz et al. 1996]. These PBPK models have led to a wide range of predictions for the amount of PCE metabolized in humans at low exposure levels, with estimates across the various models spanning an order of magnitude or more (Chiu and Ginsberg 2011).

In an attempt to reconcile these uncertainties, Chiu and Ginsberg (2011) developed a harmonized PBPK model that integrated the previous models and data. The harmonized model predicted oxidative metabolism with fairly high confidence. However, estimates of the extent of GSH conjugation in humans were substantially more uncertain, spanning more than three orders of magnitude. These predictions provide a plausible explanation for the apparently inconsistent findings among previously published models. In particular, previously conducted analyses that concluded low total metabolism (roughly 1% of PCE uptake) also assumed that TCA (derived from oxidative metabolism) represented 30–100% of total metabolism (e.g., Chen and Blancato 1989; Clewell et al. 2005; Covington et al. 2007; Qiu et al. 2010). These results are consistent with the Chiu and Ginsberg (2011) model predictions that oxidative metabolism is low in humans. On the other hand, previous analyses that concluded greater metabolism (> 20% of PCE uptake) either allowed for both oxidative metabolism and GSH conjugation, or made inferences based on the disappearance of the parent compound [e.g., Bois et al. 1990, 1996; Chiu and Bois 2006; Reitz et al. 1996; Ward et al. 1988]. These results are consistent with the Chiu and Ginsberg (2011) model predictions that the amount of GSH conjugation metabolism in humans is highly uncertain, and might be high, low, and/or highly variable.

## Carcinogenicity

*Evaluation of cancer epidemiology data*. Much of the epidemiological research has been conducted in the dry-cleaning industry, in which PCE was widely used from 1960 onward in the United States and Europe. A recent comprehensive review of 109 occupational studies with exposure measures estimated a mean exposure of 59 ppm in dry-cleaning workers based on personal measurements (Gold et al. 2008). These measures varied considerably with the type of work, however, ranging from < 10 ppm for spotters, pressers, and counter clerks to > 100 ppm for machine operators. Exposures in metal and plastic degreasing industries were also high (mean of approximately 100 ppm).

A key consideration in the evaluation of these studies was the quality (e.g., the specificity) of the exposure assessment methods. The ability of a study to identify cancer hazards is strengthened by “higher quality or higher specificity” exposure-assessment approaches that allow for a delineation of exposure potential to individual study subjects. In particular, these exposure-assessment methodologies included *a*) biological monitoring data, *b*) cohort studies using a job-exposure matrix based on historical industrial monitoring data, *c*) case–control studies using a job-exposure matrix focusing on PCE based on information on job title and tasks or duties, *d*) additional sources of information such as union records or modules for specific jobs, and *e*) studies of residential PCE exposure using a statistical model of the water distribution system to estimate the delivered dose to a study subject’s home (Table 1). Because of the variability in exposure potential within dry-cleaning occupations, less specific exposure-assessment approaches (e.g., through broader job title groups or plant- or geographically based classifications) were given less weight but were not excluded from the hazard evaluation. Other study quality considerations included a lack of support for alternative explanations for observed associations (e.g., bias or confounding).

**Table 1:** Results of epidemiological studies of PCE and bladder cancer, non-Hodgkin lymphoma, or multiple myeloma using higher quality exposure assessment methodology.

Study type/reference, population, design	Exposure surrogate	Bladder cancer RR (95% CI) [*n*]^*a*^	non-Hodgkin lymphoma RR (95% CI) [*n*]^*a*^	Multiple myeloma RR (95% CI) [*n*]^*a*^
Cohort studies
Antilla et al. 1995 (Finland): biologically monitored workers (SIR), blood PCE	Any PCE	Not reported	3.76 (0.77, 11.0) [3]	(Expected = 0.38) [0]
Boice et al. 1999 (United States): aerospace workers (SMR, RR), job-exposure matrix	Any routine exposure to PCE	0.70 (0.09, 2.53) [2]	1.70 (0.73, 3.34) [8]	0.40 (0.01, 2.25) [1]
	Duration, among those with routine or intermittent exposure to PCE:
	No routine or intermittent exposure	Not reported	1.0 (Referent) [32]	1.0 (Referent) [24]
	< 1 year		1.25 (0.43, 3.57) [4]	0.46 (0.06, 3.48) [1]
	1–4 years		1.11 (0.46, 2.70) [6]	1.13 (0.38, 3.35) [4]
	≥ 5 years		1.41 (0.67, 3.00) [10]	0.24 (0.03, 1.84) [1]
Blair et al. 2003 (United States): laundry and dry-cleaning workers (SMR), union records	Little to no PCE exposure	1.4 (0.4, 3.2) [5]	Not reported	Not reported
	Medium-to-high PCE exposure	1.5 (0.6, 3.1) [7]
Lynge et al. 2006 (Sweden, Denmark, Finland, Norway): nested case–control, census occupation codes and pension data/questionnaires	Dry-cleaner job title	1.44 (1.07, 1.93) [93]	Not studied	Not studied
	Employment duration (dry cleaner):		Not studied	Not studied
	Never	1.0 (Referent) [188]
	< 1 year	1.50 (0.57, 3.96) [6]
	2–4 years	2.39 (1.09, 5.22) [10]
	5–9 years	0.91 (0.52, 1.59) [17]
	> 10 years	1.57 (1.07, 2.29) [54]
	Unknown duration	1.97 (0.64, 6.05) [6]
Radican et al. 2008 (United States): aircraft maintenance workers (RR, internal referent), job-exposure matrix	Any PCE:
	Males	Not reported	2.32 (0.75, 7.15) [5]	1.71 (0.42, 6.91) [3]
	Females	Not reported	2.35 (0.52, 10.7) [2]	7.84 (1.43, 43.1) [2]
Seldén and Ahlborg 2011 (Sweden): dry-cleaning workers (SIR), census occupation codes, questionnaire, and company-provided data pertaining to solvent use	Any PCE:
	Males	Not reported	2.02 (1.13, 3.34) [15]	Not reported
	Females	Not reported	1.14 (0.68, 1.81) [18]	Not reported
Calvert et al. 2011 (United States): laundry and dry-cleaning workers (SMR), union employment records (PCE-only exposure based on history of solvent use by shops)	Any PCE	Not reported [0]	2.46 (0.90, 5.36) [6]	Not reported
Case–control studies
Aschengrau et al. 1993 [United States (Massachusetts)]: residential history, ordinal estimate of PCE-contaminated water from exposure model	Any PCE	1.39 (0.67, 2.91) [13]	Not studied	Not studied
	Any PCE > 90th percentile relative delivered dose	4.03 (0.65, 25.10) [4]	Not studied	Not studied
Pesch et al. 2000 (Germany): job- and task-exposure matrix	Any PCE (males):		Not studied	Not studied
	Medium exposure	1.0 (0.7, 1.5) [37]
	High exposure	1.3 (0.8, 1.7) [47]
	Substantial exposure	1.8 (1.1, 3.1) [22]
Miligi et al. 2006^*b*^, Costantini et al. 2008^*b*^ (Italy): job-exposure matrix	Any PCE:	Not studied
	Very low/low intensity		0.6 (0.3, 1.2) [18]^*c*^	Not reported [3]
	Medium/high intensity		1.2 (0.6, 2.5) [14]^*c*^	Not reported [2]
Seidler et al. 2007 (Germany): job-exposure matrix	PCE, cumulative exposure (ppm-years):	Not studied
	0		1.0 (Referent) [667]	1.0 (Referent) [33]
	> 0 to ≤ 9.1		1.1 (0.5, 2.3) [16]^*d*^	1.8 (0.5, 6.7) [3]
	> 9.1 to ≤ 78.8		1.0 (0.5, 2.2) [14]^*d*^	[0]
	> 78.8		3.4 (0.7, 17.3) [6]^*d*^	[0]
Gold et al. 2010 (United States): all jobs held >12 months, job-exposure matrix	Any PCE	Not studied	Not studied	1.5 (0.8, 2.9) [16]
	Cumulative PCE exposure (ppm-weeks):	Not studied	Not studied
	0			1.0 [164]
	1–353			0.3 (0.04, 3.0) [1]
	354–1,430			0.5 (0.1, 4.4) [1]
	1,431–4,875			1.5 (0.4, 5.4) [4]
	4,876–13,500			3.3 (1.2, 9.5) [10]
Abbreviations: RR, relative risk; SIR, standardized incidence ratio; SMR, standardized mortality ratio. ^***a***^*n* refers to the number of exposed cases. ^***b***^Both Miligi et al. (2006) and Costantini et al. (2008) are based on the Italian Multicenter Case–control Study on Hematolymphopoietic Malignancies and Exposure to Solvents and Pesticides. Miligi et al. (2006) reported ORs for non-Hodgkin lymphoma and PCE; Costantini et al. (2008) reported ORs for multiple myeloma and PCE. ^***c***^Includes patients with non-Hodgkin lymphoma and chronic lymphocytic leukemia. ^***d***^Includes patients with non-Hodgkin and Hodgkin lymphoma.				

The epidemiological evidence from cohort and case–control studies provides evidence of associations between PCE exposure and bladder cancer, non-Hodgkin lymphoma, and multiple myeloma in adults. Of these, bladder cancer and non-Hodgkin lymphoma were considered to have the strongest databases on the basis of the relative consistency of an observed association among studies with the higher quality exposure measurement and indication of increasing risk with increasing exposure among the studies using a cumulative exposure metric.

Table 1 summarizes cohort and case–control studies of bladder cancer, non-Hodgkin lymphoma, and multiple myeloma using a higher quality exposure assessment methodology; summaries of the other cancer sites can be found in the Toxicological Review (U.S. EPA 2012b). Studies of dry cleaners, launderers, and pressers used additional information to distinguish PCE-exposed workers from other workers (Blair et al. 2003; Calvert et al. 2011; Lynge et al. 2006; Pesch et al. 2000; Seldén and Ahlborg 2011). Specificity was also improved in studies in other work settings and in population-based case–control studies that used an individual-level exposure assignment (e.g., through a job-exposure matrix, PCE in blood as a biological marker, or a statistical model of water distribution to estimate a delivered PCE dose to a study subject’s home (Anttila et al. 1995; Aschengrau et al. 1993; Boice et al. 1999; Gold et al. 2010; Miligi et al. 2006; Radican et al. 2008; Seidler et al. 2007). Smoking history was considered a potential confounder only in the bladder cancer studies because it is not a known risk factor for non-Hodgkin lymphoma or multiple myeloma.

For bladder cancer, two moderate-sized (> 20 exposed cases) studies used a relatively specific exposure assessment method (Lynge et al. 2006; Pesch et al. 2000). Pesch et al. (2000) observed odds ratios (ORs) of 1.0, 1.2, and 1.8 for the medium, high, and substantial exposure categories, respectively; however, the pattern was more variable in the nested case–control study by Lynge et al. (2006), in which duration of dry-cleaning work was used as the exposure measure. Smoking is a risk factor for bladder cancer, and could also be related to TCE exposure. The case–control studies addressed this potential confounding by adjusting for smoking. The studies of dry-cleaning workers are also useful in that the study subjects are unlikely to have been exposed to other occupational bladder carcinogens. In the small studies (< 10 exposed cases) of non-Hodgkin lymphoma, an approximate doubling of risk compared with the referent population was seen (Anttila et al. 1995; Boice et al. 1999; Calvert et al. 2011; Radican et al. 2008; Seldén and Ahlborg 2011), and a relative risk of 3.4 (95% CI: 0.7, 17.3) was seen in the highest cumulative exposure group in Seidler et al. (2007). Multiple myeloma is a relatively rare type of cancer, and results for multiple myeloma are based on a smaller set of studies and fewer observed cases than those for non-Hodgkin lymphoma. The largest of these studies, with 16 exposed cases, reported an OR of 3.3 (95% CI: 1.2, 9.5) for the highest exposure group compared with the unexposed group (Gold et al. 2010). For each of the three cancer types above, the epidemiological data were considered to provide evidence suggestive of a causal association. For cancers of other sites, including esophageal, kidney, lung, liver, cervical, and breast cancer, results were more variable (data not shown). Studies of cancer in children exposed prenatally or postnatally to PCE were inadequate to allow for drawing firm conclusions (Brown Dzubow et al. 2010).

*Evaluation of experimental evidence of carcinogenicity*. There is clear evidence of PCE carcinogenicity in rodents: one oral gavage [National Cancer Institute (NCI) 1977] and two inhalation [Japan Industrial Safety and Health Association (JISA) 1993; National Toxicology Program (NTP) 1986] cancer bioassays, all in sexually mature animals. No data were available on cancer risks in experimental animals exposed to PCE during early life stages. As summarized in Table 2, a number of factors were considered in evaluating the rodent carcinogenicity findings. These included statistical analyses to adjust for survival differences and cause of death and mode-of-action analyses to inform judgments regarding human relevance of animal bioassay results and susceptible populations or life stages (U.S. EPA 2005b).

**Table 2:** Summary of factors considered in evaluating carcinogenicity findings in experimental animals.

Tumor type	Incidence (dose, sex, strain, route)	Tumor latency, severity, mortality, background rate	Toxicokinetic information	MOA information
Rat mononuclear cell leukemia	Significant increases in both sexes of F344/N (NTP 1986) and F344/DuCrj (JISA 1993) strains; dose-dependent increase in F344/DuCrj males (JISA 1993)	NTP 1986: decreased latency in females; increased severity in both sexes; background rate 56% in males, 36% in females JISA 1993: decreased latency in both sexes; background rate of ~ 20% in both sexes	No information available concerning active moiety(ies)	None hypothesized; studies demonstrating hemolysis and bone marrow toxicity in mice add some support to the biologic plausibility
Mouse hepatocellular tumors	Significant, dose-dependent increases in both sexes of B6C3F_1_ (NTP 1986) and Crj:BDF1 (JISA 1993) strains with inhalation exposures; no continued increase with increasing dose in gavage study of B6C3F_1_ strain (NCI 1977)	Decreased latency; increased mortality; increased metastases in inhalation studies; background rate of ~ 30% in males, ~ 8% in females	The metabolites TCA and DCA are mouse hepatocarcinogens, alone and in combination	Evidence is insufficient for the hypothesized MOAs evaluated: PPARα activation, mutagenicity, alterations in DNA methylation, oxidative stress secondary to cytotoxicity
Mouse hemangiomas, hemangiosarcomas	Significant, dose-dependent increases in males in one bioassay (Crj:BDF1 strain, JISA 1993)	Background rate of 2–4% in both sexes; decreased latency	No information available concerning active moiety(ies)	None hypothesized
Rat kidney tumors	Significant trend in males in one bioassay (F344/N strain, NTP 1986)	Low background rate (1/549 among historical controls for facility; ~ 0.2% in 1,968 untreated controls in the NTP program)	GSH conjugation metabolites are likely contributors to renal carcinogenicity	Evidence is insufficient for the hypothesized MOA evaluated: α2u-globulin nephropathy did not meet the U.S. EPA criteria for establishing this MOA; evidence of either *a*) cytotoxicity not associated with α2u-globulin accumulation, or *b*) peroxisome proliferation lacked specificity with regard to dose, sex and/or species; limited evidence of mutagenicity (positive Ames assays with GSH conjugation metabolites)
Abbreviations: MOA, mode of action; PPARα, peroxisome proliferator-­activated receptor α.				

**Figure 2:**
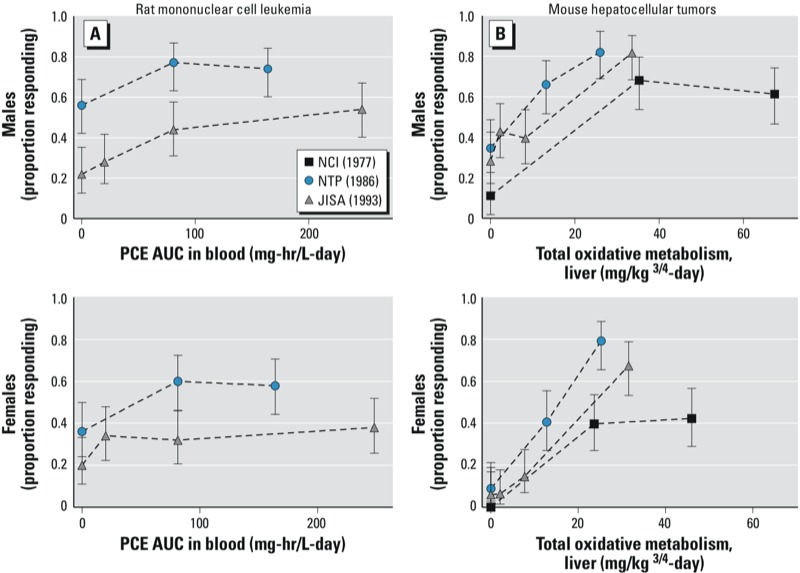
Dose–response relationships for rat mononuclear cell leukemias (*A*) and mouse hepatocellular tumors (*B*) in PCE bioassays. Three laboratories evaluated PCE in both mice and rats [oral gavage (NCI 1977); inhalation (JISA 1993; NTP 1986)]. The PBPK model of Chiu and Ginsberg (2011) was used to estimate internal dose for each site, allowing comparison of responses across routes of exposure. The best supported dose metric for mouse liver tumors was total oxidative metabolism in the liver (*B*), whereas that for rat mononuclear cell leukemia was PCE area under the curve (PCE AUC) in blood (*A*). The study in Osborne-Mendel rats (NCI 1977) was judged inconclusive because of high rates of respiratory disease and mortality with PCE and, thus, rat data from that study are not presented. Survival-adjusted responses are presented as proportion responding (incidence/number at risk).

Evaluation of rat tumors. The primary tumor finding in rats was a significant increase in the incidence of mononuclear cell leukemia in both sexes in independent inhalation bioassays using the F344/N (JISA 1993; NTP 1986) or F344/DuCrj (JISA 1993) strain (Figure 2). The NTP (1986) analyses of the PCE bioassay results revealed an increase in tumor incidence and severity in both sexes and a shortened time to death with mononuclear cell leukemia in female rats. These results were affirmed by statistical analyses performed recently by Thomas et al. (2007) who noted PCE to be 1 of only 5 of the 500 chemicals examined to produce “definitive” leukemia effects in both sexes of rats in NTP bioassays. JISA (1993) corroborated these results. There is a paucity of data in F344 rats on toxicokinetics or contributing metabolites or mechanisms to inform mode-of-action conclusions. Nonetheless, increases in hemolysis and bone marrow toxicity in NMRI mice after PCE exposure (Ebrahim et al. 2001; Marth 1987; Marth et al. 1985a, 1985b, 1989; Seidel et al. 1992)] add some support to the biologic plausibility of the observed leukemic effects (NRC 2010).

Kidney tumors, rare in male rats, were increased in a single bioassay (NTP 1986) (see Supplemental Material, Table S1). For rat kidney carcinogenesis, mechanistic data informative for the evaluation of PCE’s carcinogenic mode of action were identified (summarized in Table 2). PCE metabolites from the GSH conjugation pathway are thought to mediate mechanistic events (other than α2u-globulin nephropathy), contributing to renal carcinogenesis (Lash and Parker 2001). The GSH conjugation metabolites—TCVG, the cysteine conjugate TCVC, and the mercapturate NAcTCVC—are mutagenic in *Salmonella* tests, consistent with the observation that PCE tested positive for mutagenicity in the few studies in which products of the GSH conjugation metabolic pathway would have been generated (Dekant et al. 1986; Dreessen et al. 2003; Vamvakas et al. 1987, 1989a, 1989b). However, *in vivo* evidence of genotoxicity in the kidney is limited to reports of modest effects after intraperitoneal exposure (Mazzullo et al. 1987; Walles 1986). Evidence for α2u-globulin nephropathy did not meet the U.S. EPA’s criteria for establishing that renal tumors resulted from this mode of action (U.S. EPA 1991). In addition, evidence of either cytotoxicity not associated with α2u-globulin accumulation, or of peroxisome proliferation, lacked specificity with regard to dose, sex, and/or species.

Another rare rat tumor, brain glioma, was also increased in both sexes in a single bioassay (NTP 1986) (see Supplemental Material, Table S1). This study also reported increases in the rate of testicular interstitial cell tumors, a tumor type of high incidence in unexposed male F344 rats. Mechanistic or other data to inform the interpretation of the increases observed in rat brain and testicular tumors in the NTP bioassay were not identified.

Evaluation of mouse tumors. All three bioassays reported an increase in liver tumors in mice exposed to PCE (JISA 1993; NCI 1977; NTP 1986) (Figure 2). Statistical analyses of the gavage study (NCI 1977) revealed that the incidence of hepatocellular carcinomas or adenomas was significantly increased and that tumor latency was significantly decreased. A significant association between increased mortality and PCE dose was seen, with liver tumors found in many mice that died before scheduled termination. Inhalation exposure to PCE induced significant, dose-related increases in the incidence of hepatocellular adenomas or carcinomas in both sexes of B6C3F_1_ (NTP 1986) and Crj:BDF1 (JISA 1993) mice. The incidence of hepatocellular carcinomas that metastasized to the lungs was also significantly increased in the inhalation studies.

The mouse liver tumor evaluation also considered data on the hepatocarcinogenicity of the PCE metabolites TCA and DCA. The primary urinary oxidative metabolite in rodents and humans, TCA, significantly increased the incidence of liver tumors in male and female B6C3F_1_ mice exposed via drinking water for 52–104 weeks (Bull et al. 1990, 2002; DeAngelo et al. 2008; Herren-Freund et al. 1987; Pereira 1996). Liver tumor incidence increased with increasing concentrations of TCA in drinking water (Bull et al. 1990, 2002; DeAngelo et al. 2008; Pereira 1996). Tumors in TCA-exposed animals developed rapidly, and significant increases were evident in less-than-lifetime studies of ≤ 82 weeks. In addition, TCA was hepatocarcinogenic in mice when coadministered in the drinking water for 52 weeks with the PCE metabolite DCA (Bull et al. 2002). DCA alone also causes liver cancer in mice (Bull et al. 1990; Daniel et al. 1992; DeAngelo et al. 1999; Herren-Freund et al. 1987).

The issue of whether TCA can solely account for the hepatocarcinogenicity of PCE was addressed using toxicokinetic analyses because the hepatocarcinogenic potencies of TCA and PCE have not been directly compared in a single rodent bioassay. The U.S. EPA’s analysis found that a wide range of possible contributions of TCA to PCE carcinogenicity, from as little as 12% to as much as 100%, is consistent with the available data (U.S. EPA 2012b). A more precise quantitative estimate of the relative contribution of TCA to PCE-induced liver tumors requires an appropriately designed study to better control for experimental variability in kinetics (e.g., dosing patterns in drinking water, metabolism, bioavailability) and dynamics (e.g., background tumor rates).

For hepatocarcinogenesis, a key issue was whether evidence for peroxisome proliferation is sufficient to establish the peroxisome proliferator-activated receptor α (PPARα)–activation mode of action. The relevance to humans of tumors induced by this mode of action has been debated (e.g., Corton 2008; Guyton et al. 2009; Klaunig et al. 2003; Rusyn and Corton 2012; Rusyn et al. 2006). For instance, a dissenting opinion provided in the NRC peer review (NRC 2010, Appendix B) stated that

the weight of evidence strongly favors a key role of PPARα activation in tetrachloroethylene-induced hepatocarcinogenesis in mice; furthermore, this mode of action lacks relevance for human hepatocarcinogenesis.

However, the NRC peer-review committee as a whole did not support these conclusions, stating in rebuttal that many gaps in knowledge remain with regard to the hepatocarcinogenic mechanisms of PCE. PCE-specific experiments have provided evidence that peroxisomal markers are increased, but at dose levels (i.e., 1,000 mg/kg/day) exceeding those causing liver toxicity, proliferation, or carcinogenicity (Odum et al. 1988; Philip et al. 2007). In particular, Philip et al. (2007) reported that CYP4A, a marker of PPARα-activation, was only increased in Swiss Webster mice at the highest PCE dose (1,000 mg/kg/day) and at the earliest (7 days), but not later, time points. In contrast, the study reported a robust dose-dependent proliferative response that persisted for 14–30 days postexposure at 150-, 500-, and 1,000-mg/kg/day levels of PCE. Liver toxicity and repair has been reported at lower doses in other studies in the B6C3F_1_ strain [e.g., at 100 mg/kg/day (Schumann et al. 1980)]. Moderate increases in peroxisome proliferation have been reported in rats (Odum et al. 1988), a species insensitive to PCE hepatocarcinogenicity. In total, these findings indicate that the modest peroxisome proliferative response to PCE may lack specificity with respect to species, tissue, and dose. The temporal sequence of events also remains to be established. Given these limitations, the database of PCE-specific studies was judged insufficient to demonstrate a causative role of this effect in hepatocarcinogenesis by PCE (U.S. EPA 2012b). PCE and/or its metabolites have been shown to induce a number of other mechanistic events that may also contribute to carcinogenicity, including mutagenicity, alterations in DNA methylation, and oxidative stress (U.S. EPA 2012b).

In addition to mouse liver tumors, hemangiosarcomas or hemangiomas of the liver, spleen, fat, and subcutaneous skin were reported in male mice in one inhalation study (JISA 1993) (see Supplemental Material, Table S2). This mouse tumor type was not reported in the NCI oral gavage bioassay (NCI 1977), and no increase was reported in the NTP inhalation bioassay (NTP 1986). Mechanistic or other data to inform the interpretation of this tumor type were not identified.

*Conclusions on carcinogenic hazard*. Supported by the analyses described above, and following the U.S. EPA’s *Guidelines for Carcinogen Risk Assessment* (U.S. EPA 2005a), PCE is characterized as “likely to be carcinogenic to humans” (U.S. EPA 2012b). This characterization is based on suggestive evidence of carcinogenicity in epidemiological studies and conclusive evidence that the administration of PCE, either by ingestion or by inhalation to sexually mature rats and mice, increases tumor incidence (JISA 1993; NCI 1977; NTP 1986). The specific carcinogenic active moiety(ies) and mode(s) of action are not fully characterized, and the hypothesis that mutagenicity contributes to the PCE carcinogenesis has not been ruled out, particularly for kidney carcinogenicity. No data were available on cancer risks in animals exposed to PCE during early life stages.

## Noncancer Toxicity

The U.S. EPA’s analysis identified the central nervous system, kidney, liver, immune and hematologic systems, and development and reproduction, as target organs of PCE toxicity (U.S. EPA 2012c). Although sufficient for hazard identification, the supporting evidence for several end points was limited for characterizing the relationship of effect with dose at low exposures. Neurotoxicity was supported by a considerable database of human, animal, and mechanistic studies. In addition, neurological effects were generally observed at lower PCE concentrations compared with other noncancer health effects. Further, both the 2004 peer consultation workshop (U.S. EPA 2004) and the 2010 NRC peer review (NRC 2010) affirmed the conclusion that neurotoxicity is a sensitive end point because these effects were observed at lower concentrations and had substantial evidential support. The human and animal neurotoxicity findings supporting the U.S. EPA’s conclusions concerning PCE neurotoxicity are summarized below.

*Neurotoxicity*. Human studies regarding neurotoxicological hazard. The three primary neurological domains most consistently associated with subchronic or chronic PCE exposure in human studies were vision, visuospatial memory, and neuropsychological function (e.g., reaction time). Occupational and residential exposure studies support an association of visual deficits after chronic PCE exposure. Deficits in color vision, relative to unexposed study participants, were observed in an occupational study (Cavalleri et al. 1994) and in a residential study (Schreiber et al. 2002). In a longitudinal follow-up study to Cavalleri et al (1994), Gobba et al (1998) reported that there was a worsening in the color vision in workers (self-comparison) who were exposed to higher levels of PCE. In the dry-cleaning facilities, color vision deficits, reported as a color confusion index (CCI) (Iregren et al. 2002), were significantly greater in exposed workers than in unexposed controls, with mean CCI scores of 1.143 and 1.108, respectively (*p* = 0.03) (Cavalleri et al. 1994). An additional 6% (*p* < 0.01) deterioration in mean CCI score relative to their previous score (reported by Cavalleri et al. 1994) was seen in workers exposed to increasing concentrations of PCE (median, 1.67–4.35 ppm) 2 years later in a follow-up study of the same worker population (Gobba et al. 1998). Schreiber et al. (2002) reported lower CCI scores, in comparison with a nonexposed residential group, for adult and child residents living within a close proximity to dry cleaners, with mean CCI scores of 1.33 in exposed study participants and 1.20 in controls (*p* = 0.26). In the same study, no difference in CCI scores was observed in day-care workers working in a day-care center next to a dry cleaner in comparison with day-care workers in buildings with no PCE exposure. Two studies did not observe changes in color vision with PCE exposure, but they were limited by having no exposure characterization (Sharanjeet-Kaur et al. 2004) or by using less sensitive color vision testing (Nakatsuka et al. 1992). In addition, deficits in visual contrast sensitivity relative to unexposed study participants were reported in the two residential populations living or working in buildings co-located with dry cleaners (Schreiber et al. 2002; Storm et al. 2011). There was a decreasing trend (*p* < 0.05) for visual contrast sensitivity in the residential population achieving the maximum contrast sensitivity score at 6 cycles per degree, with 28.3% in the referent group versus 8.3% in the highest exposed (> 100 μg/m^3^) group (Storm et al. 2011).

Associations between exposure and visuospatial memory were also reported in each of the studies that examined this measure in humans. These associations (increased response times or cognition errors) were reported in occupational (Echeverria et al. 1994, 1995; Seeber 1989) and residential (Altmann et al. 1995) studies related to dry-cleaning PCE exposure. In the occupational studies (Echeverria et al. 1994, 1995; Seeber, 1989), cognition errors ranged from 4 to 30% at exposures of 12–23 ppm, depending on the subtest that was used. In the residential study (Altmann et al. 1995), visual memory and cognitive function scores were 15% lower in individuals with a mean exposure of 0.7 ppm PCE compared with unexposed individuals. No studies specifically examined visuospatial memory in children.

For neuropsychological function, two studies reported 10–20% increases in simple reaction time, in comparison with an unexposed group, in PCE-exposed occupational (Ferroni et al. 1992) and residential (Altmann et al. 1995) settings. However, another occupational study reported a 16% improvement in simple reaction time in comparison with unexposed individuals (Lauwerys et al. 1983).

Animal studies of neurotoxicological effects. Animal studies of subchronic PCE exposure also observed changes in visual function, cognitive function, and reaction time. In rats, acute inhalation exposure to PCE resulted in changes in visual-evoked potentials (Boyes et al. 2009; Mattsson et al. 1998). In one subchronic exposure study (Mattsson et al. 1998), a significant increase in amplitude and latency was observed in one peak of the visual-evoked potential responses from a flash stimulus at 5,424 mg/m^3^, but histopathological lesions were not observed in the examination of central and peripheral brain structures (e.g., visual cortex, optic nerve) of the visual system.

Significant decrements in the motor activity domain as measured by increased reaction time, increased number of false alarms, and decreased trial completions in a signal detection task (measures of decreased attention) were reported at ≥ 6,782 mg/m^3^ in an acute exposure study in rats (Oshiro et al. 2008). In addition, deficits in operant tasks that test cognitive performance were seen in rats and mice after acute oral (Warren et al. 1996) and intraperitoneal (Umezu et al. 1997) exposures to PCE. These findings support evidence of deficits in cognition and memory associated with PCE exposure in humans. However, no animal studies to date have evaluated the persistence of cognitive performance deficits from acute or chronic PCE exposure.

Observed changes in brain weight and levels of DNA, RNA, and neurotransmitters in experimental animals are consistent with evidence of neurobehavioral effects of PCE exposure in humans. Brain DNA, RNA and protein levels, as well as lipid composition, were altered after PCE inhalation. Changes were observed in the cerebellum, the hippocampus, and the frontal cortex in sexually mature animals (Rosengren et al. 1986; Savolainen et al. 1977a, 1977b; Wang et al. 1993) as well as after gestational exposure (Kyrklund and Haglid 1991; Nelson et al. 1979).

*Conclusions regarding noncancer hazard*. The U.S. EPA’s analysis identified the central nervous system, kidney, liver, immune and hematologic systems, and development and reproduction, as target organs of PCE toxicity (U.S. EPA 2012c). Neurotoxicity was identified as among the most sensitive outcomes, occurring at low exposures. The assessment of the neurotoxicity studies drew conclusions through an examination of affected domains (e.g., cognition, vision, motor activity). Human and experimental animal studies provided complementary evidence regarding the association of neurobehavioral deficits and PCE exposure. Studies of humans exposed by inhalation suggest that chronic PCE exposure can result in decrements in vision, visuospatial memory, and, possibly, other aspects of cognition and neuropsychological function, including reaction time. Animal studies provide substantial support for associations of PCE exposure with effects in these domains of neurotoxicity.

## Summary

PCE is a widespread contaminant that is present in ambient air, indoor air, soil, drinking water, and groundwater. Once exposed, humans and laboratory animal species rapidly absorb PCE. PCE is then distributed to tissues via the systemic circulation, metabolized, and excreted primarily in breath as unchanged PCE or CO_2_, or in urine as metabolites. The role of metabolism in the toxicity of PCE was informed by the development and application of an updated PBPK model (Chiu and Ginsberg 2011). Low oxidative metabolism was predicted in humans, whereas GSH conjugation metabolism is more uncertain and may be high, low, and/or highly variable. These PBPK model predictions informed the extent and nature of metabolism in different target tissues, and the extrapolation across species and routes of exposure.

Following the U.S. EPA’s *Guidelines for Carcinogen Risk Assessment* (U.S. EPA 2005a), PCE was characterized as “likely to be carcinogenic to humans” by all routes of exposure. This characterization is based on suggestive evidence of carcinogenicity in epidemiological studies and conclusive evidence that the administration of PCE, either by ingestion or by inhalation to sexually mature rats and mice, increases tumor incidence (JISA 1993; NCI 1977; NTP 1986).

Neurotoxicity is identified as a sensitive outcome that follows either oral or inhalation exposure to PCE in humans and experimental animals. Associations between exposure and neurotoxic outcomes have been reported by human controlled exposure, occupational, and residential studies as well as experimental animal studies, providing evidence that PCE exposure results in visual changes, increased reaction time, and decrements in cognition.

The U.S. EPA’s analysis, approaches, and conclusions are consistent with multiple sets of peer-reviewer recommendations (U.S. EPA 2012a). In addition, the use of evidence tables, narrative syntheses, and other aspects of the assessment approach were in accord with later NRC recommendations for improving IRIS assessments (NRC 2011). The International Agency for Research on Cancer recently classified PCE as probably carcinogenic to humans (Group 2A) on the basis of sufficient evidence in animals and limited evidence in humans, consistent with the U.S. EPA’s conclusion (Guha et al. 2012). Finally, studies of the health effects of PCE published since the U.S. EPA’s assessment continue to report associations with neurological outcomes, including studies of illicit drug use (Aschengrau et al. 2011); mental illness (Aschengrau et al. 2012); visual effects (Getz et al. 2012); visuospatial functioning, learning and memory, motor, attention, and mood (Janulewicz et al. 2012); and Parkinson disease (Goldman et al. 2012). Similarly, recent analyses of PCE exposure and cancer continue to add support for human tumor sites identified in the Toxicological Review (Christensen et al. 2013; Lipworth et al. 2011; Ruder et al. 2013; Vizcaya et al. 2013; Vlaanderen et al. 2013; 2014).

## Supplemental Material

(270 KB) PDF
