# Supplementary material for: Human Health Effects of Tetrachloroethylene: Key Findings and Scientific Issues
Source: Environ Health Perspect. 2014 Feb 14;122(4):325–34. doi: 10.1289/ehp.1307359 (PMC3984230; doi:10.1289/ehp.1307359)
Supplement: (270 KB) PDF [file ehp.1307359.s001.pdf]

Environmental Health Perspectives doi:10.1289/ehp.1307359

Supplemental Material

Human Health Effects of Tetrachloroethylene: Key Findings and Scientific Issues

Kathryn Z. Guyton, Karen A. Hogan, Cheryl Siegel Scott, Glinda S. Cooper, Ambuja S. Bale, Leonid Kopylev, Stanley Barone Jr., Susan L. Makris, Barbara Glenn, Ravi P. Subramaniam, Maureen R. Gwinn, Rebecca C. Dzubow, and Weihsueh A. Chiu

Table of Contents **Page**

Table S1. Tumor incidence in adult rats exposed to tetrachloroethylene **2**

Table S2. Tumor incidence in adult mice exposed to tetrachloroethylene **3**

References **4**

Table S1. Tumor incidence in adult rats exposed to tetrachloroethylene.

Bioassay	Sex	Administered Exposure	Mononuclear cell leukemia[a]	Kidney adenoma or carcinoma	Testis interstitial cell tumor	Brain glioma
NTP (1986) F344/N rats; inhalation: 6 h/d, 5 d/wk, 104 wk	Males	0	28/50[b] (56)[c]	1/49 (2)[c]	35/50 (70)[c]	1/50 (2)[c]
		200 ppm	37/48 (77)	3/47 (6)	39/47 (83)	0/48 (0)
		400 ppm	37/50 (74)	4/50 (8)	41/50 (82)	4/50 (8)
	Females	0	18/50 (36)[c]	0/50 (0)	N/A	1/50 (2)
		200 ppm	30/50 (60)	0/49 (0)		0/50 (0
		400 ppm	29/50 (58)	0/50 (0)		2/50 (4)
JISA (1993) F344/DuCrj rats; inhalation: 6 h/d, 5 d/wk, 104 wk	Males	0	11/50 (22)[c]	1/50 (2)	47/50 (94)	2/50 (4)
		50 ppm	14/50 (28)	2/50 (4)	46/50 (92)	0/50 (0)
		200 ppm	22/50 (44)	1/50 (2)	45/50 (90)	0/50 (0)
		600 ppm	27/50 (54)	2/50 (4)	48/50 (96)	0/50 (0)
	Females	0	10/50 (20)[c]	1/50 (2)	N/A	0/50 (0)
		50 ppm	17/50 (34)	0/50 (0)		0/50 (0)
		200 ppm	16/50 (32)	0/50 (0)		1/50 (2)
		600 ppm	19/50 (38)	1/50 (2)		0/50 (0)

[a]Reflects the number of animals with MCL reported under "multiple organs," spleen, or liver. [b]Fractions represent survival-adjusted incidences. [c]$p \leq 0.05$:Poly-3 trend test (Portier and Bailer, 1989; Bailer and Piegorsch, 1997).

Table S2. Tumor incidence in adult mice exposed to tetrachloroethylene.

Bioassay	Sex	Administered exposure	Total oxidative metabolism, liver[a]	Hepatocellular adenomas or carcinomas	Hemangiomas or hemangio-sarcomas[b]
NCI (1977)[c] $B6C3F_1$ mice Gavage: 5d/wk, 78 wk	Males	Vehicle	0	2/18(11)[d]	None reported[e]
		536 mg/kg-day	35.3	32/47 (67)	
		1,072 mg/kg-day	67.4	27/44 (60)	
	Females	Vehicle	0	0/20 (0)[c]	None reported
		386 mg/kg-day	23.7	19/48 (40)	
		772 mg/kg-day	46.0	19/48 (40)	
NTP (1986) $B6C3F_1$ mice Inhalation: 6 h/d, 5 d/wk, 104 wk	Males	0 ppm	0	17/49 (35)[c]	1/49 (2)
		100 ppm	13.2	31/47 (66)	0/49 (0)
		200 ppm	26.0	41/50 (82)	0/50 (0)
	Females	0 ppm	0	4/45 (9)[c]	0/48 (0)
		100 ppm	12.8	17/42 (40)	3/50 (6)
		200 ppm	25.3	38/48 (76)	0/50 (0)
JISA (1993) Crj:BDF1 mice Inhalation: 6 h/d, 5 d/wk, 104 wk	Males	0 ppm	0	13/46(28)[c]	1/46 (2)[c]
		10 ppm	2.3	21/49 (43)	1/49 (2)
		50 ppm	8.3	19/48 (40)	5/48 (10)
		250 ppm	33.6	40/49 (82)	5/49 (10)
	Females	0 ppm	0	3/50 (6)[c]	1/50 (2)
		10 ppm	2.1	3/47 (6)	0/47 (0)
		50 ppm	7.8	7/48 (15)	2/49 (4)
		250 ppm	31.6	33/49 (67)	3/50 (6)

[a]In mg/kg$^{3/4}$-day. [b]Reported as hemangioendotheliomas by JISA (1993). The term has been updated to hemangioma (benign) or hemangiosarcoma (malignant). [c]Initial gavage doses of 450 or 900 mg/kg-day (males) and 300 or 600 mg/kg-day (females) were adjusted several times during the course of the study; listed exposures are TWA daily doses through Week 78. Surviving animals were observed up to study termination in Week 90. [d]$p < 0.05$: Cochran-Armitage test for trend (NCI), Life atable test (NTP, JISA). [e]None reported: Individual animal data were not available, and summary data were not reported for this tumor type.

References

JISA (Japan Industrial Safety and Health Association). 1993. Carcinogenicity study of tetrachloroethylene by inhalation in rats and mice. Hadano, Japan. Available at: http://www.epa.gov/iris/supdocs/0106index.html

NCI (National Cancer Institute). 1977. Bioassay of tetrachloroethylene for possible carcinogenicity. NCI-CGTR-13; DHEW Publication No. (NIH) 77-813. Bethesda, MD:National Institutes of Health.

NTP (National Toxicology Program). 1986. Toxicology and carcinogenesis studies of tetrachloroethylene (perchloroethylene) (CAS no. 127-18-4) in F344/N rats and B6C3F1 mice (inhalation studies). NTP TR 311. Research Triangle Park, NC:U.S. Department of Health and Human Services, National Toxicology Program.

Piegorsch WW, Bailer AJ. 1997. Statistics for Environmental Biology and Toxicology. London: Chapman and Hall.

Portier CJ, Bailer AJ. 1989. Testing for increased carcinogenicity using a survival-adjusted quantal response test. Fundam Appl Toxicol. 12(4):731-7.
